# Comparative Efficacy of Er:YAG Laser and Dental Turbine in Pediatric Dentistry: A Systematic Review

**DOI:** 10.3390/children13020258

**Published:** 2026-02-12

**Authors:** Gianna Dipalma, Angelo Michele Inchingolo, Paola Nardelli, Lucia Casamassima, Danilo Ciccarese, Paolo De Sena, Francesco Inchingolo, Andrea Palermo, Grazia Marinelli, Alessio Danilo Inchingolo

**Affiliations:** 1 Department of Life Science, Health, and Health Professions, Link Campus University, 00165 Rome, Italy; g.dipalma@unilink.it; 2 Department of Biomedical, Surgical and Dental Sciences, School of Dentistry, University of Milan, 20100 Milan, Italy; angelo.inchingolo@unimi.it; 3 Department of Interdisciplinary Medicine, University of Bari “Aldo Moro”, 70124 Bari, Italy; paola.nardelli@uniba.it (P.N.); lucia.casamassima@uniba.it (L.C.); danilo.ciccarese@uniba.it (D.C.); paolo.desena@uniba.it (P.D.S.); graziamarinelli@live.it (G.M.); alessiodanilo.inchingolo@uniba.it (A.D.I.); 4 Department of Experimental Medicine, University of Salento, 73100 Lecce, Italy; andrea.palermo@unisalento.it

**Keywords:** pediatric dentistry, Er:YAG laser, dental turbine, caries removal, primary teeth, patient comfort, minimally invasive dentistry

## Abstract

Aim: This systematic review compared erbium lasers (Er:YAG/Er,Cr:YSGG) with conventional rotary instruments (dental turbine/high-speed handpiece) for caries removal and cavity preparation in pediatric dentistry, focusing on patient-centered outcomes and short-term restorative performance. Methods: Following PRISMA guidance, PubMed, Scopus, and Web of Science were searched for studies published between January 2010 and November 2025. Eligible studies were in vivo/human investigations in children with carious primary teeth comparing erbium laser versus rotary instrumentation. Results: Eleven studies met the inclusion criteria. Across the included trials, erbium laser treatment was consistently associated with reduced intraoperative pain and improved comfort, often accompanied by lower anxiety indicators and higher child acceptance compared with rotary preparation. Several studies also reported a reduced need for local anesthesia in the laser group. In contrast, operative time was generally longer with erbium lasers than with turbines. When restorations were evaluated, clinical performance and short-term success (up to 12 months) were comparable between laser- and bur-prepared cavities, with no consistent disadvantages observed for laser preparation. Conclusions: Overall, erbium lasers appear to be a clinically effective and child-friendly alternative to conventional turbines, offering superior patient comfort while maintaining comparable short-term restorative outcomes, albeit at the cost of longer procedure duration.

## 1. Introduction

In contemporary clinical practice, the management of dental caries in juvenile patients represents a significant challenge [1,2,3]. Successful treatment requires not only effective removal of carious tissue but also careful consideration of the emotional and behavioral responses of young children during dental procedures [4,5,6]. For decades, conventional high-speed rotary instruments, commonly referred to as dental turbines, have been regarded as the gold standard for cavity preparation because of their efficiency and rapid cutting ability [5,7]. Nevertheless, their use is frequently associated with discomfort, vibration, noise, and potential thermal irritation, factors that may increase anxiety and reduce cooperation in pediatric patients during treatment [4,8].

**Figure 1:**
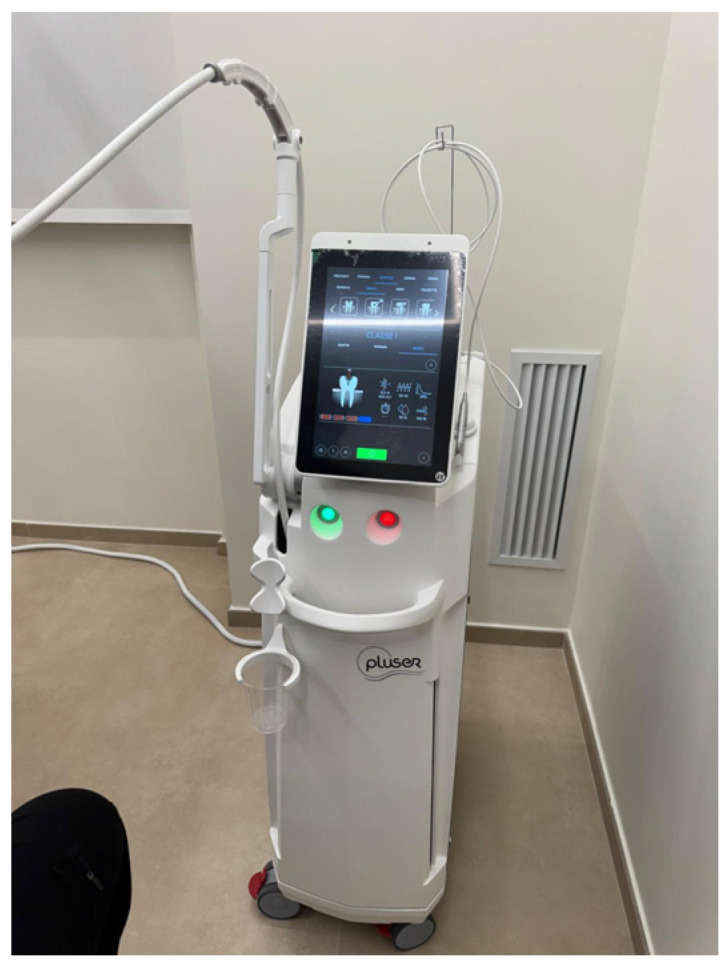
Modern Er:YAG dental laser system for advanced clinical procedures.

In response to these limitations, technological advances have promoted the development of alternative approaches aimed at enhancing minimally invasive dentistry and improving children’s acceptance of dental procedures [9,10,11]. Among these, the erbium-doped yttrium aluminum garnet (Er:YAG) laser has emerged as a promising option, as it allows selective removal of carious tissue while minimizing thermal damage and improving patient comfort (Figure 1) [12,13].

**Figure 2:**
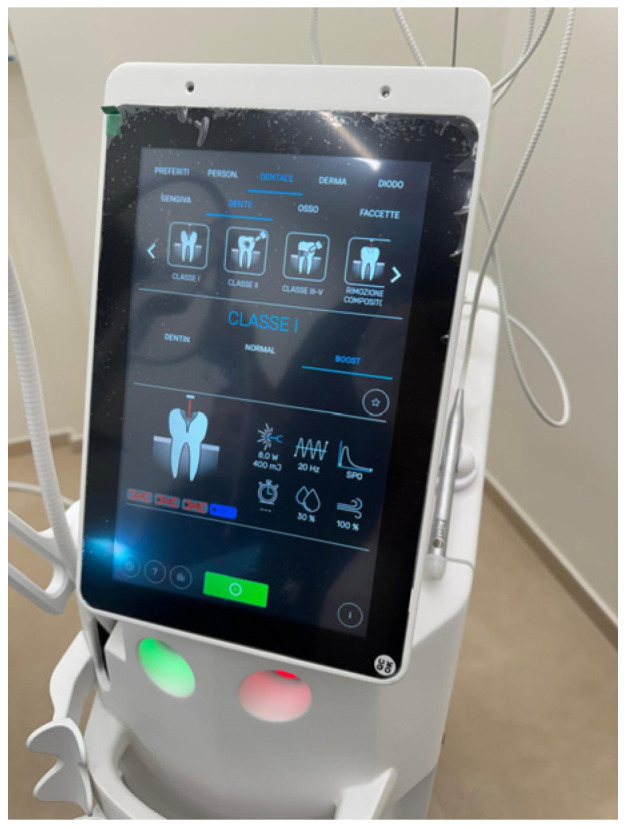
The screen displays specific clinical settings including power (8.0 W), frequency (20 Hz), and various treatment categories like Class I-V cavities and composite removal.

The Er:YAG laser operates at a wavelength of 2940 nm, which coincides with a strong absorption peak of both hydroxyapatite and water, the principal components of enamel and dentin [14,15,16]. This characteristic enables a photothermal ablation mechanism based on micro-explosive events that permit precise and selective removal of demineralized dentin while limiting collateral effects [17,18,19]. The ablation process is primarily driven by rapid vaporization of water within dental hard tissues (Figure 2) [1,2,3,20,21,22,23,24,25,26,27,28,29,30,31,32].

Because laser energy is absorbed superficially, tissue cutting occurs with minimal thermal diffusion, thereby reducing the risk of pulpal overheating and often decreasing the need for local anesthesia [2,33,34,35]. Furthermore, the absence of high-frequency noise and mechanical vibration contributes to enhanced patient comfort, and several studies have reported that children show greater tolerance toward laser-assisted procedures compared with conventional rotary instrumentation [20]. These advantages align closely with the clinical objectives of pediatric dentistry and the principles of minimally invasive treatment, particularly in early childhood care [32,36,37,38].

Conversely, the dental turbine remains widely used because of its long-standing clinical familiarity, ease of handling, and high operational speed [39,40]. By mechanically abrading dental tissues through high-speed rotating burs, turbines often generate discomfort, as well as characteristic noise and vibration, which may be poorly tolerated by young patients [23,40]. Although turbines are highly effective in rapidly excavating carious lesions, their lack of selectivity between infected and affected dentin may result in unnecessary removal of sound tooth structure [39,40]. In addition, rotary instrumentation produces a smear layer that can interfere with adhesive bonding and restoration longevity, and frictional heat generation necessitates continuous water cooling [41,42,43,44,45,46,47,48]. Despite these disadvantages, dental turbines remain the most accessible and cost-effective instruments in both private and public pediatric dentistry settings [27,41,49,50,51,52].

For these reasons, the comparison between Er:YAG lasers and conventional dental turbines is particularly relevant in pediatric dentistry, where minimal invasiveness, treatment acceptance, and behavioral management are essential considerations [10,53,54]. An increasing number of randomized controlled trials and comparative studies have evaluated the effectiveness of Er:YAG lasers in primary dentition, reporting favorable outcomes in terms of patient acceptance, cavity preparation quality, and restoration integrity [52,55,56,57]. Compared with children treated using rotary burs, those treated with Er:YAG lasers often exhibit reduced anxiety, improved cooperation, and lower levels of perceived pain [51,55,58,59,60]. Moreover, laser-prepared cavities demonstrate advantageous micromorphological characteristics, such as increased surface roughness and the absence of a smear layer, which may enhance adhesive performance [16,17,18,19,52,54].

Despite these reported benefits, the clinical superiority of laser technology over conventional turbines remains a topic of debate [38,61,62]. Although Er:YAG lasers can achieve restoration durability comparable to that obtained with burs and superior patient-reported outcomes, evidence from systematic reviews and meta-analyses indicates that laser-assisted procedures often require longer operative times and involve higher acquisition and maintenance costs [36,37,48,58,63]. Additionally, effective laser use demands specific training and careful adjustment of operating parameters [64,65,66,67,68,69,70,71,72,73,74]. As a consequence, considerable variability exists in the adoption of laser technology across different pediatric dental settings [75,76,77,78,79,80,81,82,83,84,85].

Therefore, the present systematic review aims to evaluate whether caries removal using erbium-based lasers (Er:YAG and Er,Cr:YSGG) leads to improved clinical and patient-centered outcomes in pediatric dentistry when compared with conventional rotary instruments (high-speed dental turbines). Specifically, this review focuses on intraoperative pain, the need for local anesthesia, anxiety levels, child behavior and cooperation, procedure duration, and the short- to medium-term clinical performance of restorations in primary teeth.

## 2. Materials and Methods

### 2.1. Protocol and Registration

The current systematic review followed the PRISMA guidelines (Preferred Reporting Items for Systematic Reviews and Meta-Analyses) and International Prospective Register of Systematic Review Registry procedures (full ID:1274463) (see Supplementary Materials) [86].

### 2.2. Search Process

A search of the following databases was conducted in September 2025: PubMed, Web of Science (WOS), and Scopus were examined from 1 January 2010 to 30 November 2025 in order to search articles of the last 15 years (Table 1). The search strategy was created by combining terms relevant to the study’s purpose. The search strategy was designed by integrating terms aligned with the study’s objectives. In the advanced search queries applied across the databases (with the complete search terms provided in Appendix A), the keywords were combined using Boolean operators to capture concepts relevant to the study’s aims (Table 1): (“Er:YAG” OR “Er,Cr:YSGG” OR “erbium laser”) AND (“dental caries” OR caries OR “cavity preparation” OR “caries removal”) AND (rotary OR “air rotor” OR turbine OR “high speed handpiece”).

### 2.3. Inclusion and Exclusion Criteria

The reviewers worked in groups to assess all relevant studies that were evaluated. Studies were included if they met the following criteria:

- Open-access articles;
- Studies written in English;
- Studies conducted in vivo or on humans;
- Case–control studies, cohort studies, and randomized controlled trials (RCTs);
- Studies published in the last 15 years.

Eligible investigations comprised randomized controlled trials, cohort studies, and case–control studies involving pediatric patients with carious primary teeth, in which caries removal or cavity preparation performed using erbium-based lasers (Er:YAG or Er,Cr:YSGG) was directly compared with conventional rotary instruments (high-speed dental turbine). Only studies published within the predefined time frame were considered.

Exclusion criteria comprised review articles, case reports or case series, letters or editorials, in vitro and animal studies, investigations conducted in adult populations, and studies not directly comparing erbium laser techniques with rotary instrumentation in pediatric dentistry.

### 2.4. PICO Question

The PICO format is a framework used in qualitative research to structure clinical research questions. The PICO addressed the question: “In children with dental caries requiring restorative treatment, does caries removal using an erbium laser, compared with conventional rotary instruments (high-speed handpiece), reduce intraoperative pain and the need for local anaesthesia, improve child behaviour/cooperation, and provide comparable operative time and clinical outcomes of the restoration?”.

The PICO question is developed as follows:

I. Population (P): Children with carious primary teeth.
II. Intervention (I): Caries removal using an erbium laser (e.g., Er:YAG or Er,Cr:YSGG).
III. Comparison (C): Conventional caries removal with rotary instruments/high-speed handpiece (turbine).
IV. Outcome (O): Intraoperative pain, need for local anaesthesia, child behaviour/cooperation during treatment, and operative time (and, secondarily, clinical quality of the restoration).

### 2.5. Data Processing

Four independent reviewers (L.C., D.C., P.D.S, and P.N.) assessed the included studies’ quality using selection criteria, methods of outcome evaluation, and data analysis. This enhanced ‘risk of bias’ tool additionally provides quality standards for selection, performance, detection, reporting, and other biases. All differences were settled through conversation or collaboration with other researchers (G.D., A.P., A.D.I. and A.M.I.). The reviewers screened the records according to the inclusion and exclusion criteria. The 1.202 selected articles were downloaded into “Zotero 6.0.36” for organization and analysis.

## 3. Results

### 3.1. Selection and Characteristics of the Study

**Figure 3:**
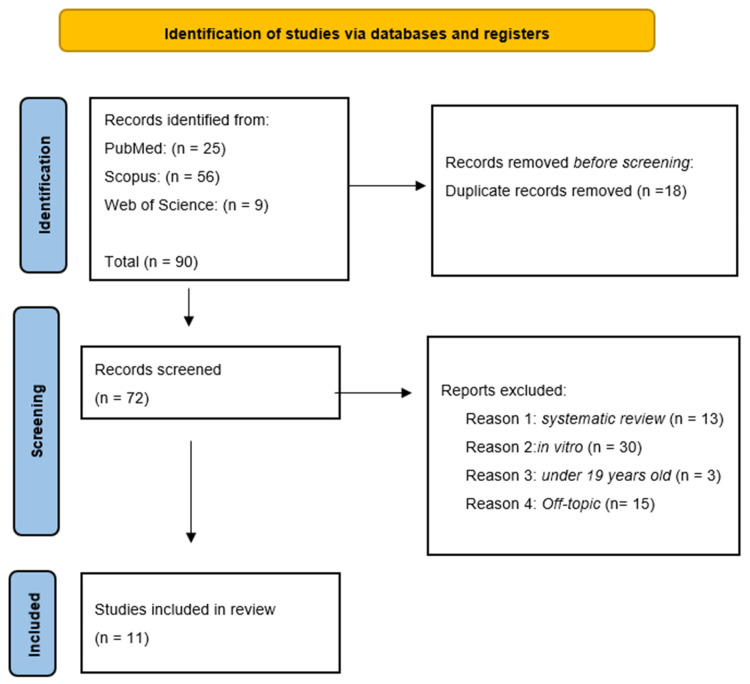
PRISMA flowchart.

This PRISMA (Preferred Reporting Items for Systematic Reviews and Meta-Analyses) diagram (Figure 3) illustrates a rigorous and systematic selection process to ensure that only relevant studies were included in the final review. In total, 90 records were identified through database searching: PubMed (n = 25), Scopus (n = 56) and Web of Science (n = 9). After the removal of 18 duplicate records, 72 records were screened by title and abstract. Of these, 61 were excluded for the following reasons: systematic reviews (n = 13), in vitro studies (n = 30), studies conducted in adult populations (n = 3), and off-topic articles not related to erbium laser caries removal in paediatric patients (n = 15). Finally, 11 studies met the inclusion criteria and were included in the qualitative review. The selection process and summary of included records are illustrated in Figure 3, while the characteristics of the selected studies are presented in Table 2.

### 3.2. Quality Assessment and Risk of Bias of Included Articles

The methodological quality of the included manuscripts was assessed by a reviewer (L.C.) using risk-of-bias tools tailored to study design. Specifically, the Cochrane RoB 2 (Risk of Bias 2) tool was applied to the randomized trials (Table 3): Belcheva (2014), Valério (2016), Korkut (2018), Johar (2019), Alia (2020), Abdrabuh (2023a), Abdrabuh (2023b), Xu (2024), and Milc (2025). RoB 2 evaluates five domains: (1) bias arising from the randomization process, (2) bias due to deviations from intended interventions, (3) bias due to missing outcome data, (4) bias in measurement of the outcome, and (5) bias in selection of the reported result, and assigns an overall risk-of-bias judgement categorized as “Low risk of bias,” “Some concerns,” or “High risk of bias.”

In contrast, for the non-randomized/quasi-experimental studies, Bohari (2012) and El-Dehna (2021), the ROBINS-I (Risk Of Bias In Non-randomized Studies of Interventions) tool was used (Table 4). ROBINS-I assesses seven domains: (1) bias due to confounding, (2) bias in selection of participants into the study, (3) bias in classification of interventions, (4) bias due to deviations from intended interventions, (5) bias due to missing data, (6) bias in measurement of outcomes, and (7) bias in selection of the reported result, followed by an overall risk-of-bias judgement categorized as “Low,” “Moderate,” “Serious,” “Critical,” or “No information.”

Overall, the RoB 2 assessment (Table 3) indicated that the randomized trials mostly fell within the “some concerns” range, largely driven by issues commonly affecting pediatric dental trials with subjective outcomes (e.g., incomplete reporting of key randomization details and/or limited blinding of outcome assessment for pain/anxiety measures). In a smaller subset of studies, the overall judgement increased to “high risk of bias,” typically when at least one domain was rated high risk, most frequently related to outcome measurement and/or missing outcome data. For the two non-randomized studies assessed with ROBINS-I (Table 4), Bohari (2012) was judged to have an overall moderate risk of bias, mainly reflecting expected confounding and limited information on some methodological safeguards, whereas El-Dehna (2021) showed an overall serious risk of bias, primarily driven by confounding (non-random allocation features inherent to the split-mouth procedure) and potential deviations from intended interventions. Using two complementary instruments allowed a consistent appraisal of internal validity across heterogeneous designs, ensuring that each study was evaluated with the tool most appropriate to its methodological structure.

## 4. Discussion

Minimally invasive approaches for caries removal have gained increasing attention in pediatric dentistry, driven by the need to reduce pain, anxiety, and behavioral management challenges while maintaining effective clinical outcomes. Among these approaches, erbium-based lasers (Er:YAG and Er,Cr:YSGG) have been proposed as alternatives to conventional rotary instruments because they can ablate hard dental tissues with minimal vibration, noise, and thermal damage. The studies included in this discussion collectively examine whether these theoretical advantages translate into tangible clinical and behavioral benefits for children undergoing caries removal.

### 4.1. Effectiveness of Caries Removal and Cavity Quality

Across the included studies, erbium lasers demonstrated comparable effectiveness to conventional rotary instruments in removing carious tissue. Bohari et al. and Valério et al. showed that Er:YAG laser excavation achieved reductions in DIAGNOdent values similar to those obtained with air rotor burs, indicating thorough removal of infected dentin. Similarly, Johar et al. confirmed complete caries removal with both Er,Cr:YSGG laser and conventional handpieces using caries-detection dyes [21,46,53].

Beyond effectiveness, several authors highlighted differences in cavity morphology. Valério et al. reported that laser-prepared cavities were more conservative, with less removal of sound tooth structure, aligning with the principles of minimally invasive dentistry [46]. Importantly, studies focusing on restoration outcomes, such as Abdrabuh et al. (2023) and El-Dehna et al., demonstrated that cavity preparation with lasers did not compromise restoration quality [41,54]. Using modified USPHS or Ryge criteria, both laser- and bur-prepared cavities showed high Alpha scores for marginal adaptation, anatomical form, and secondary caries, even after follow-up periods of up to 12 months. These findings were further corroborated by Xu et al., who reported similar restoration success rates and no increased risk of tooth fractures with laser use [44].

Nevertheless, it should be noted that the included studies employed highly heterogeneous laser parameters, including variations in energy output, pulse frequency, and water/air spray settings. This heterogeneity may have influenced both cutting efficiency and biological response, potentially contributing to variability in reported outcomes across studies. The absence of standardized laser protocols limits direct comparability and makes it difficult to determine whether observed differences are attributable to the laser technique itself or to specific parameter configurations.

### 4.2. Operative Time and Clinical Efficiency

A consistent finding across nearly all studies was the longer operative time associated with laser-based caries removal. Bohari et al., Valério et al., Johar et al., and Milc et al. all reported significantly longer treatment durations when using erbium lasers compared with rotary instruments [21,46,53,62]. In particular, Milc et al. quantified this difference, noting that laser preparation required approximately 2.5 times longer than turbine-based techniques [62].

Despite this limitation, most authors emphasized that the increased operative time did not negatively affect clinical feasibility. Bohari et al. noted that laser treatment was still faster than chemomechanical methods, while Xu et al. argued that the slower cutting efficiency was offset by improved patient cooperation and reduced need for anesthesia [44,53]. However, it is plausible that differences in laser settings—such as lower energy or frequency chosen to maximize comfort—may partly explain the prolonged operative times observed in some trials. Collectively, these findings suggest that while operative time remains a practical drawback, it may be clinically acceptable in pediatric settings where behavioral management is a priority.

### 4.3. Pain Perception and Need for Local Anesthesia

Pain reduction emerged as one of the most consistently reported advantages of erbium laser use. Studies employing validated pain scales—including Wong–Baker FACES, VAS, FLACC, and universal pain assessment tools—uniformly demonstrated lower pain scores with laser treatment. Belcheva et al., Korkut et al., and Milc et al. reported striking differences, with a substantial proportion of children experiencing little to no pain during laser procedures [62,79,87]. Notably, Milc et al. observed complete elimination of pain (VAS = 0) in the laser group.

Several studies also reported a reduced need for local anesthesia. Xu et al. and Abdrabuh et al. (2023) found that significantly fewer children requested anesthesia during laser treatment compared with conventional drilling. Although El-Dehna et al. and Alia et al. did not always find statistically significant differences in pain scores, both studies still described a clear clinical trend favoring laser-assisted procedures; these findings underscore the potential of erbium lasers to enhance patient comfort and reduce pharmacological intervention in pediatric dentistry.

It is important to emphasize, however, that pain perception is inherently subjective, particularly in pediatric populations. Most studies relied on self-reported scales or observer-based assessments, which may be influenced by age, cognitive development, previous dental experiences, and contextual factors. This subjectivity represents a relevant limitation and may introduce variability or bias in the estimation of true analgesic differences between laser and rotary techniques.

### 4.4. Anxiety, Behavioral Response, and Patient Acceptance

Anxiety reduction and improved behavior represent another key advantage of laser-assisted caries removal. Studies assessing anxiety through physiological measures, such as pulse rate, and behavioral scales consistently favored laser use. Alia et al. demonstrated that rotary instruments significantly increased pulse rate, whereas laser treatment caused only minimal changes [88]. Similarly, Abdrabuh et al. (2023) reported lower anxiety scores and better cooperation with laser preparation using Venham’s Dental Anxiety Scale [32].

**Figure 4:**
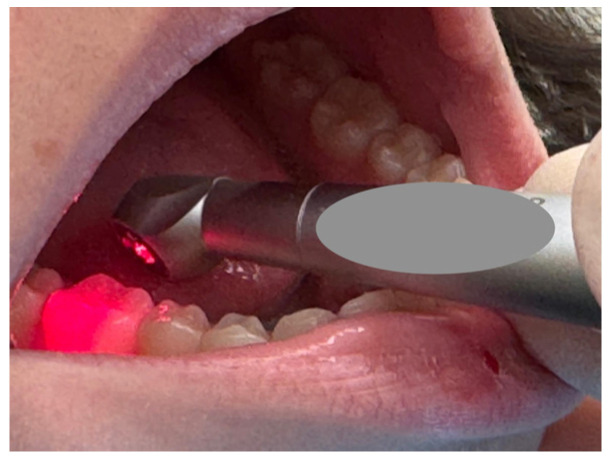
A close-up view of a dental procedure, showing a dental instrument emitting a red light being applied to a molar inside a patient’s mouth.

Behavioral improvements were often attributed to the absence of vibration, pressure, and high-pitched noise. Bohari et al., Korkut et al., and Xu et al. all emphasized that these factors contributed to improved child cooperation and a more positive dental experience. Importantly, preference data further supported these observations: Alia et al. reported that 57% of children preferred the laser for future treatments, even when pain differences were minimal [44,53,79,88]. This suggests that acceptance is influenced not only by pain, but also by the overall sensory experience of the procedure (Figure 4).

Nonetheless, anxiety-related outcomes share similar methodological limitations to pain measures, as they are often based on subjective or semi-objective scales. Emotional state, environmental context, and operator–child interaction may all affect these assessments, warranting cautious interpretation of the magnitude of anxiety reduction attributed to laser use.

### 4.5. Economic Considerations and Clinical Feasibility

Beyond clinical and behavioral outcomes, the adoption of erbium laser technology must also be considered in light of economic sustainability and feasibility, particularly within public healthcare systems. Although higher acquisition and maintenance costs, longer operative times, and the need for specific training are frequently mentioned as disadvantages, these aspects were only marginally addressed in the included studies. In high-volume pediatric settings, such as public dental services, these factors may limit widespread implementation. However, potential indirect benefits—such as improved cooperation, reduced need for local anesthesia, and fewer interrupted treatments—may partially counterbalance these costs. Dedicated cost-effectiveness analyses are therefore required to clarify whether the improved patient-centered outcomes associated with laser use translate into long-term practical advantages in routine clinical practice.

## 5. Conclusions

This systematic review aimed to determine whether erbium-based lasers (Er:YAG and Er,Cr:YSGG) offer advantages over conventional rotary instruments for caries removal and cavity preparation in pediatric dentistry, with a focus on patient-centered outcomes and short-term restorative performance. The findings of the included studies consistently support the objectives outlined in the Introduction.

Erbium lasers demonstrated superior performance in reducing intraoperative pain, decreasing the need for local anesthesia, and improving child comfort, cooperation, and anxiety management—directly addressing the clinical and behavioral challenges central to pediatric dental care. Although operative time was generally longer with laser use, this drawback did not compromise restoration quality or short-term success, meeting the objective of evaluating both clinical efficiency and restorative outcomes. Moreover, restorations placed in laser-prepared cavities showed comparable integrity to those prepared with traditional rotary instruments over short-term follow-up.

Overall, the evidence indicates that erbium lasers represent a clinically effective and child-friendly alternative to conventional rotary tools, aligning with the goals stated in the Introduction to enhance minimally invasive, behavior-oriented dental care for pediatric patients. Future research with standardized protocols, larger samples, and extended follow-up will be essential to clarify long-term outcomes and further refine clinical decision-making.

## 6. Future Limitations (Integrated with Study Limitations)

Despite the promising clinical and patient-centered benefits reported for erbium lasers, several limitations of the present review must be acknowledged. First, the included studies exhibited substantial methodological heterogeneity, particularly in study design (parallel vs. split-mouth), laser type (Er:YAG vs. Er,Cr:YSGG), device settings, operative protocols, restorative materials, and outcome measures. This variability limits direct comparability among trials, also preventing a quantitative meta-analysis. Additionally, many studies enrolled relatively small samples and provided only short follow-up periods—often up to 6–12 months—reducing the ability to assess long-term restoration performance and treatment stability.

Blinding of operators and participants was inherently unfeasible, introducing potential performance and detection bias, especially for subjective outcomes such as pain, anxiety, and child cooperation. In several studies, incomplete reporting of randomization procedures, allocation concealment, and pre-specified outcomes contributed to uncertainties during risk-of-bias assessment. Furthermore, heterogeneity in evaluation methods and outcome measures made standardization challenging.

Beyond the limitations of the current evidence base, future research should aim to overcome these issues by conducting well-designed, adequately powered randomized controlled trials with standardized laser parameters, harmonized outcome measures, and longer follow-up periods. Additional investigations should also explore cost-effectiveness, operator learning curves, and practical implementation challenges, which remain understudied yet critical for integrating laser technology into routine pediatric dental practice.

## Figures and Tables

**Table 1:** Indicators for database searches.

Article screening strategy	Keywords: “Er:YAG, Er,Cr:YSGG, erbium laser, dental caries, caries, cavity preparation, caries removal, rotary, air rotor, turbine, high speed handpiece”
	Boolean Indicators: OR and AND
	Timespan: 1 January 2010 to 30 November 2025
	Electronic databases: PubMed; Scopus; WOS.

**Table 2:** Analysis of the studies included in the Discussion section from 2010 to 2025.

Authors	Type of the Study	Patients	Material and Methods	Aim of the Study	Results
Bohari et al., 2012 [53]	In vivo comparative clinical study	120 primary teeth in children aged 5–9	Four caries-removal techniques were compared: air rotor, Carisolv™, Papacarie^®^, and Er:YAG laser. Efficacy was assessed by DIAGNOdent values, efficiency by operative time, and patient discomfort by FLACC scale.	To compare efficacy, efficiency, and pain/discomfort associated with conventional, chemomechanical, and erbium-laser caries removal in primary teeth.	Air rotor and laser were most effective; air rotor was fastest. Laser and chemomechanical methods caused significantly less pain and greater comfort. Laser provided efficacy comparable to conventional drilling with better patient acceptance.
Belcheva et al., 2014 [87]	Randomized clinical comparative study	90 children aged 6–12	Two treatment groups: Er:YAG laser (2940 nm) vs. conventional rotary instruments, both used without anesthesia. Pain was assessed post-treatment using the universal pain assessment tool (Wong–Baker FACES + VAS scale).	To evaluate and compare pediatric pain perception during cavity preparation using Er:YAG laser and rotary instruments.	Laser-treated children reported significantly lower pain. Most experienced low pain levels, and severe pain was rare. The laser was considerably more comfortable than conventional drilling, representing a suitable alternative for pediatric restorative procedures.
Valério et al., 2016 [46]	Clinical comparative study	42 children initially (age 6–10 years)	Compared Er:YAG laser vs. high-speed handpiece for caries removal. Evaluated efficiency, operative time, DIAGNOdent readings, and clinical/behavioral responses.	To compare the effectiveness and characteristics of caries removal using Er:YAG laser versus conventional rotary instruments in pediatric patients.	Both methods effectively removed carious dentin. Laser preserved more sound tissue and improved patient comfort but required more time. Clinical results were comparable, supporting the laser as a viable alternative for pediatric dentistry.
Korkut et al., 2018 [79]	Split-mouth clinical study	120 children (8–12 years)	Caries removal on paired permanent molars using Er:YAG laser (Lightwalker; 2940 nm; 300 mJ/20 Hz for enamel; 250 mJ/4 Hz for dentin) and conventional high-/low-speed rotary instruments. No anesthesia used. Pain evaluated via Wong–Baker FACES scale.	To evaluate and compare children’s pain perception during caries removal performed with Er:YAG laser versus conventional rotary instruments.	Laser treatment caused significantly less pain. More children reported “no hurt,” and none reported severe pain. The Er:YAG laser was more comfortable and acceptable, representing a preferable alternative to conventional rotary instruments for pediatric patients.
Johar et al., 2019 [21]	In vivo split-mouth clinical study	25 children (6–10 years)	Each child had two matched primary teeth with class I lesions. One tooth treated with Er,Cr:YSGG laser (Waterlase; 6 W, 25 Hz; 60:40 water/air), the other with air rotor handpiece (400,000 rpm). Pain measured with WBFPRS and VAS. Operative time recorded. Caries-detection dye used to assess efficacy.	To compare pain perception, operative time, and efficacy of caries removal using Er,Cr:YSGG laser versus air rotor handpiece in primary teeth.	Children experienced significantly less pain with the laser; 80% preferred it. Laser required more time but was equally effective at caries removal. The Er,Cr:YSGG laser is a comfortable and acceptable alternative to the air rotor for pediatric patients.
Alia et al., 2020 [88]	Randomized controlled split-mouth clinical trial	30 children (6–12 years)	60 molars treated using airotor in one quadrant and Er,Cr:YSGG laser (Waterlase iPlus, 2780 nm) in the other. Anxiety measured by pulse oximeter; pain by Wong–Baker scale. Children asked preference after both treatments.	To compare pain, anxiety, and acceptance of Er,Cr:YSGG laser vs conventional rotary cavity preparation in children.	Airotor caused significantly higher anxiety; laser caused less anxiety and slightly less pain. 57% preferred laser. The laser proved more acceptable and calmer for pediatric patients.
Abdrabuh et al., 2023 [54]	Randomized controlled split-mouth clinical trial	40 children (9–12 years)	Class I cavities in primary molars prepared either with Er:YAG laser (2940 nm) or conventional rotary bur; restorations placed using a resin-based composite system; clinical evaluation after 1 year according to Ryge (USPHS) criteria	To compare the integrity and clinical performance of restorations in primary teeth prepared with Er:YAG laser versus conventional rotary instruments	Er:YAG laser cavity preparation showed restoration integrity comparable to conventional bur preparation after one year, with no significant differences between techniques, supporting its clinical effectiveness as a minimally invasive alternative in pediatric dentistry
El-Dehna et al., 2021 [41]	In vivo split-mouth clinical study	20 children (4–7 years), 40 primary teeth	Class I occlusal cavities in primary molars prepared either with erbium laser or conventional turbine; restorations placed using high-viscosity glass ionomer (Equia Forte) or bulk-fill composite; assessment of pain, operative time, need for anesthesia, and clinical performance using modified USPHS criteria over follow-up	To compare laser and conventional caries removal techniques in children and to evaluate the clinical performance of bulk-fill composite and high-viscosity glass ionomer restorations	Laser preparation reduced pain perception and discomfort compared with conventional drilling but required longer operative time; both restorative materials showed excellent and comparable clinical performance, supporting laser use as a minimally invasive option in pediatric dentistry
Abdrabuh et al., 2023 [32]	Randomized controlled split-mouth clinical trial	35 children (9–12 years)	Bilateral occlusal carious primary molars treated in a split-mouth design; cavities prepared either with Er:YAG laser (2940 nm) or conventional rotary instruments; anxiety assessed using pulse oximetry and Venham’s scale; pain assessed using Wong–Baker FACES Pain Rating Scale; need for local anesthesia recorded	To compare pain perception and anxiety levels in children during caries removal using Er:YAG laser versus conventional rotary techniques	Er:YAG laser caries removal significantly reduced pain, anxiety, and demand for local anesthesia compared with conventional drilling, improving child cooperation and comfort during treatment
Xu et al., 2024 [44]	Split-mouth clinical comparative study	78 children (5–10 years) (156 carious molars)	Each child received turbine treatment on one maxillary molar and Er:YAG laser on the contralateral molar; outcomes included anesthesia use, pain, hypersensitivity, tooth fracture, anxiety/cooperation, operative time, and 12-month success rate.	To compare Er:YAG laser and traditional dental turbine for pediatric caries removal in terms of comfort, cooperation, operative efficiency, and short-term restoration outcomes.	Er:YAG laser significantly reduced pain, hypersensitivity, anesthesia need, and anxiety, while improving cooperation; operative time was longer, but 12-month success rates were comparable, supporting laser as a more comfortable yet clinically effective option.
Milc et al., 2025 [62]	Randomized clinical trial	33 children (66 deciduous teeth), aged 3–8 years	Two groups: Er:YAG laser vs. dental turbine. Recorded operative time; pain assessed with VAS. Laser parameters: 230 mJ for enamel, 120–150 mJ for dentin, 10–20 Hz. No local anesthesia used; included healthy first-time pediatric patients with deep caries in unicuspid primary teeth	To determine optimal Er:YAG laser settings for caries removal in deciduous teeth and compare operative time and pain levels with conventional turbine treatment.	Er:YAG laser significantly reduced pain (VAS = 0) and improved comfort and cooperation, though requiring longer preparation time; its minimally invasive and quiet operation reduces anxiety and may decrease need for anesthesia, making it superior to conventional turbine therapy in pediatric patients.

**Table 3:** Tabular summary of the RoB 2 assessment for 9 studies, evaluated across 5 domains.

AUTHORS AND YEARS	D1	D2	D3	D4	D5	Overall
Belcheva et al., 2014	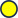	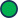	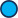	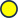	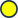	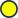
Valério et al., 2016	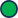	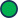	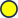	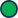	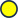	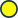
Korkut et al., 2018	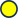	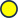	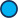	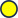	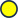	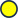
Johar et al., 2019	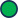	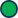	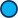	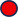	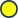	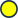
Alia et al., 2020	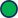	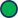	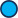	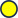	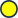	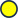
Abdrabuh et al., 2023(a)	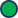	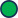	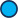	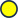	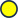	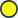
Abdrabuh et al., 2023(b)	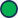	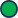	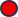	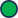	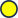	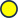
Xu et al., 2024	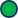	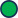	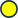	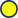	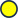	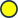
Milc et al., 2025	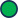	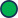	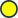	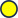	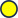	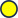
**Domains:**				**Judgement:**		
D1: Bias arising from the randomization process				Moderate risk		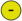
D2: Bias due to deviations from intended interventions				Low risk		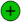
D3: Bias due to missing outcome data				No Information		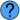
D4: Bias in the measurement of the outcome				High risk		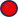
D5: Bias in the selection of the reported result						
Overall: Overall risk of bias						

**Table 4:** Tabular summary of the ROBINS-I assessment for 2 studies, evaluated across 7 domains.

AUTHORS AND YEARS	D1	D2	D3	D4	D5	D6	D7 Overall
Bohari et al., 2012	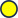	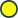	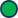	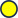	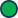	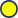	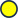
El-Dehna et al., 2016	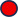	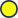	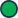	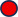	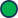	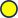	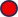
Domains:				Judgement:			
D1: Bias due to confounding				Moderate risk			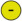
D2: Bias in selection of participants into the study				Low risk			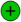
D3: Bias in classification of interventions				No Information			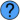
D4: Bias due to deviations from intended interventions				High Risk			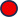
D5: Bias due to missing data				Serious Risk			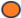
D6: Bias in measurement of outcomes							
D7: Bias in selection of the reported result							
Overall: Overall risk of bias							

## Data Availability

Data are contained within the article.

## Supplementary Materials

The following supporting information can be downloaded at: https://www.mdpi.com/article/10.3390/children13020258/s1, PRISMA 2020 Checklist.

## Abbreviations

The following abbreviations are used in this manuscript:

ADA	American Dental Association
DIAGNOdent	Laser fluorescence device for caries detection
Er,Cr:YSGG	Erbium, Chromium-doped Yttrium Scandium Gallium Garnet
Er:YAG	Erbium-doped Yttrium Aluminum Garnet
FLACC	Face, Legs, Activity, Cry, Consolability scale
MeSH	Medical Subject Headings
PRISMA	Preferred Reporting Items for Systematic Reviews and Meta-Analyses
PICO	Population, Intervention, Comparison, Outcome
RCTs	Randomized Controlled Trials
ROBINS-I	Risk Of Bias In Non-Randomized Studies of Interventions
RoB 2	Risk of Bias 2 tool
USPHS	United States Public Health Service criteria
VAS	Visual Analogue Scale
WBFPRS	Wong–Baker FACES Pain Rating Scale
WOS	Web of Science

## Appendix A

### Appendix A.1. PubMed Query String

(“Er:YAG”[All Fields] OR “Er,Cr:YSGG”[All Fields] OR “erbium laser”[All Fields]) AND (“dental caries”[MeSH Terms] OR “dental caries”[All Fields] OR caries[All Fields] OR “cavity preparation”[All Fields] OR “caries removal”[All Fields]) AND (“rotary”[All Fields] OR “air rotor”[All Fields] OR turbine[All Fields] OR “high speed handpiece”[All Fields]).

### Appendix A.2. Scopus Query String

TITLE-ABS-KEY (“Er:YAG” OR “Er,Cr:YSGG” OR “erbium laser”) AND TITLE-ABS-KEY (“dental caries” OR caries OR “cavity preparation” OR “caries removal”) AND TITLE-ABS-KEY (rotary OR “air rotor” OR turbine OR “high speed handpiece”).

### Appendix A.3. Web of Science Query String

ALL=(“Er:YAG” OR “Er,Cr:YSGG” OR “erbium laser”) AND ALL=(“dental caries” OR caries OR “cavity preparation” OR “caries removal”) AND ALL=(“rotary” OR “air rotor” OR turbine OR “high speed handpiece”).
